# Biphasic CAPA-IVM Improves Equine Oocyte Quality and Subsequent Embryo Development Without Inducing Genetic Aberrations

**DOI:** 10.3390/ijms26125495

**Published:** 2025-06-08

**Authors:** Muhammad Fakhar-I-Adil, Daniel Angel-Velez, Emin Araftpoor, Qurratul Ain Amin, Mohamed Hedia, Marcel Bühler, Kris Gevaert, Björn Menten, Ann Van Soom, Susana Marina Chuva de Sousa Lopes, Dominic Stoop, Chloë De Roo, Katrien Smits, Björn Heindryckx

**Affiliations:** 1 Ghent-Fertility and Stem Cell Team (G-FaST), Department for Reproductive Medicine, Department of Human Structure and Repair, Ghent University Hospital, Corneel Heymanslaan 10, 9000 Ghent, Belgium; muhammad.fakhariadil@ugent.be; 2 Department of Internal Medicine, Reproduction and Population Medicine, Ghent University, 9820 Merelbeke, Belgium; daniel.angelvelez@ugent.be (D.A.-V.); qurratulain.amin@ugent.be (Q.A.A.); mohamed.hedia@ugent.be (M.H.); ann.vansoom@ugent.be (A.V.S.); katrien.smits@ugent.be (K.S.); 3 VIB-UGent Center for Medical Biotechnology, Vlaams Instituut voor Biotechnologie (VIB), 9052 Ghent, Belgium; emin.araftpoor@ugent.be (E.A.); marcel.buhler@ugent.be (M.B.); kris.gevaert@ugent.be (K.G.); 4 Department of Biomolecular Medicine, Ghent University, 9052 Ghent, Belgium; 5 Theriogenology Department, Faculty of Veterinary Medicine, Cairo University, Giza 12211, Egypt; 6 Center for Medical Genetics, Ghent University Hospital, 9000 Ghent, Belgium; bjorn.menten@ugent.be; 7 Department of Anatomy and Embryology, Leiden University Medical Center, 2333 ZA Leiden, The Netherlands; s.m.chuva_de_sousa_lopes@lumc.nl; 8 Department for Reproductive Medicine, Women’s Clinic, Ghent University Hospital, Corneel Heymanslaan 10, 9000 Ghent, Belgium; dominic.stoop@uzgent.be (D.S.); chloe.deroo@uzgent.be (C.D.R.)

**Keywords:** CAPA-IVM, equine embryos, in vitro maturation, oocyte proteomics, equine euploidy

## Abstract

In vitro maturation (IVM) of oocytes retrieved from ovum pick-up (OPU) or ovarian tissue (OT) is a standard approach for patients with specific conditions where prior hormonal stimulation is contraindicated. However, the developmental competence of oocytes matured in vitro is still inferior to that of oocytes matured in vivo. Capacitation IVM (CAPA-IVM) includes an extra step of pre-maturation culture (PMC) with c-type natriuretic peptide (CNP) as a meiotic arrestor to better synchronize cytoplasmic and nuclear maturity in oocytes by allowing the cytoplasm additional time to acquire essential components critical for optimal competency. This study aims to evaluate the effect of CAPA-IVM on equine oocyte quality and developmental competence. Immature cumulus–oocyte complexes (COCs) were retrieved from slaughterhouse ovaries and matured in vitro either in CAPA-IVM (short 6 h, long 24 h pre-maturation) or standard IVM. Mature oocytes from each group were analyzed for calcium-releasing potential (*n* = 52) and single-oocyte proteomics (*n* = 44), and embryo development (*n* = 229) was assessed after fertilization with piezo-drilled intracytoplasmic sperm injection (ICSI). Genetic analysis of developed blastocysts (*n* = 41) was performed to detect chromosomal aberrations. Our findings demonstrate that CAPA-IVM of equine COCs yields significantly higher maturation rates than controls. Moreover, short CAPA-IVM with six hours pre-maturation culture showed substantially higher embryo development potential than the control group (20/69 vs. 9/63, respectively). Genetic analysis revealed a high euploidy rate in equine blastocysts regardless of the maturation conditions. Live calcium imaging of the fertilized oocytes demonstrated that the majority of oocytes displayed non-continuous calcium oscillation patterns, irrespective of maturation conditions. Single-oocyte proteomics reveals a comparable proteomic landscape between mature oocytes subjected to short CAPA-IVM and standard IVM. However, we identified four enriched gene sets with positive enrichment scores after short CAPA-IVM, related to cytoskeleton regulation, ribosomal function, and cytosolic components. Our findings indicate that CAPA-IVM holds the potential to improve oocyte quality and competence in horses. However, further fine-tuning of culture conditions would benefit the effective use of these IVM systems. Moreover, given that the mare serves as an excellent model for human reproduction, the molecular trends identified in this study could provide valuable insights for advancing human artificial reproductive technologies.

## 1. Introduction

In vitro maturation (IVM) of oocytes is a standard approach for patients with contraindications to conventional hormonal stimulations. This includes cases such as polycystic ovary syndrome (PCOS), ovarian hyperstimulation syndrome (OHSS), resistant ovary syndrome (ROS), and malignancies requiring immediate intervention [1]. It provides an alternative to conventional stimulation protocols where oocytes are matured in vivo before retrieval [1]. Additionally, IVM is also a routine procedure in other species, such as cattle and especially horses, where ovarian hormonal stimulation is often ineffective [2]. Furthermore, oocytes from euthanized mares or slaughterhouse ovaries also require a prior step of IVM for the successful generation of equine embryos in vitro [3], a process crucial for maximizing genetic potential and breed preservation.

IVM involves resumption of the meiosis following retrieval of immature cumulus-oocyte complexes (COCs) from ovarian tissue without or with minimal hormonal stimulation. Although these oocytes achieve nuclear maturity following IVM, their cytoplasmic maturation—which involves attaining molecular modifications such as maternal mRNA regulation, organelle redistribution, epigenetic modifications, cytoskeletal rearrangements, and calcium signaling regulation necessary for successful fertilization and embryo development [4]—remains unclear. Additionally, the developmental competence of oocytes matured in vitro is still inferior to that of oocytes matured in vivo [5,6].

Since certain modifications such as organelle redistribution, cytoskeletal remodeling, maternal mRNA accumulation, and epigenetic changes occur in vivo after the activation and progression of the follicle before the luteinizing hormone (LH) wave, the term oocyte capacitation [7] or oocyte pre-maturation [8] is used to indicate this phase of oocyte growth. However, during this phase, the follicular milieu is vital in maintaining meiotic arrest and enabling timely meiosis resumption. C-type natriuretic peptide (CNP)-derived elevation in oocyte cAMP level arrests the oocyte in the germinal vesicle (GV) phase by inhibiting maturation-promoting factor (MPF) activity through cAMP-dependent kinase A [9]. Nonetheless, a drop in oocyte and follicular cGMP levels occurs due to LH surge, which leads to upregulation of halted phosphodiesterase3 (PDE3) activity, which in turn resumes the meiosis by lowering intraoocyte cAMP [9,10,11]. Further changes in the cytoplasm involving redistribution of organelles also happen during this phase [12]. Before ovulation, the oocyte must acquire essential components required for normal fertilization and early embryo development [13].

To recapitulate these conditions in vitro, several in vitro pre-maturation strategies using meiotic arrestors have been investigated in various species, including humans, mice, goats, cattle, pigs, sheep, and horses [14,15]. The aim of this effort is to enhance the cytoplasmic maturity of oocytes, to support embryo development better. Usually, oocytes undergoing IVM are collected from follicles of varying sizes, resulting in a diverse range of GV chromatin configurations. These variations influence the timing of germinal vesicle breakdown (GVBD) [16]. Consequently, upon spontaneous resumption of meiosis, asynchronous maturation of the nucleus and cytoplasm occurs across the cohort of oocytes, complicating the precise timing required for subsequent procedures like sperm injection. Notably, the primary objective of the original biphasic IVM study was to attain synchronized meiotic progression in pig oocytes during IVM [17].

Most pre-maturation strategies in biphasic IVM mainly focus on regulating cyclic nucleotide levels in cumulus–oocyte complexes (COCs) using specific pharmacological agents. Specifically, PDE3 inhibitors, such as cilostamide, milrinone, or Org9935, have been used to modulate cyclic nucleotide levels [18,19]. Alternatively, exogenous dibutyryl cAMP (dbcAMP) supplementation [17,20] or pharmacological induction of cAMP using forskolin [21] or invasive adenylate cyclase [22] have been employed to achieve elevated cAMP levels and to better mimic the in vivo environment.

The recognition of C-type natriuretic peptide (CNP) as a physiological meiotic arrestor prompted the reassessment of the previous understanding on oocyte pre-maturation [23] and led to the basis of a CNP-mediated pre-IVM system [24]. Furthermore, assessment of CNP-based IVM systems in various species such as pigs, goats, sheep, mice, and humans also showed encouraging results [15]. As reviewed by Gilchrist et al. [15], CNP-based pre-maturation enhances oocyte quality and developmental potential by delaying premature meiotic resumption, preserving cumulus–oocyte communication, and promoting oocyte growth, metabolic activity, and chromatin remodeling. Moreover, the clinical application of CNP-based CAPA-IVM has shown promising results in human patients where prior ovarian stimulation is not feasible, such as gynecological cancer [25], and PCOS patients with variable antral follicle counts [26,27,28,29,30,31]. It can further form the basis of fertility preservation for individuals with the indication of ovariectomy, such as transgender men and oncology patients. Moreover, ongoing efforts of in vitro gametogenesis [32,33,34] can benefit from this technique to improve final oocyte quality.

In equine artificial reproductive technology (ART), the inefficacy of ovarian hormonal stimulation [2] strongly emphasizes oocyte maturation as an essential step for in vitro embryo production. Moreover, holding equine oocytes at room temperature is a common practice that facilitates the oocyte’s transportation and further convenient scheduling for subsequent procedures [35]. For the first time, Choi et al. [36,37] assessed holding equine oocytes in a holding medium containing meiotic inhibitors or not and reported comparable embryo outcomes to direct IVM. These findings highlight the resilience of equine oocytes to withstand a holding step without compromising developmental potential [38]. Since holding oocytes is a common practice, incorporating effective pre-IVM strategies could fulfill this purpose and enhance embryo outcomes. Considering insights and advancements in human CAPA-IVM, we sought to investigate the use of CNP as a meiotic arrestor to improve equine oocyte quality and developmental competence. Moreover, calcium imaging and single-oocyte proteome analysis were performed on matured oocytes with the aim of identifying key proteins affecting oocyte potential. So far, only a few studies have been published on oocyte pre-maturation culture in horses, which showed compromised [37] or improved developmental potential [36,39,40], mainly affected by the stage of immature oocytes used.

This study is the first to evaluate a CNP-based pre-maturation culture in the context of equine IVM and to apply single-oocyte proteomics to refine oocyte holding and maturation protocols in ART. The mare serves as an excellent translational model due to key physiological and developmental similarities to humans [41,42]. In addition to its value as a model species, the horse holds considerable economic, cultural, and sporting significance, motivating the ongoing development of innovative reproductive strategies to enhance genetic potential and safeguard valuable breeds. Importantly, advances in equine ARTs not only benefit the equine industry but may also provide reciprocal insights that contribute to improving human reproductive medicine.

## 2. Results

### 2.1. Long CAPA-IVM Increases Maturation Rates While Showing a Trend of Compromised Developmental Competency

In Experiment 1 (3 replicates), a total of 199 COCs were cultured in both groups. Our results show that long CAPA-IVM oocytes (*n* = 61) with 24 h of pre-maturation culture yielded significantly higher maturation rates (69% vs. 51%, *p* = 0.017) than the control group (*n* = 138). Oocyte survival rates were not affected by this extended maturation culture (70% vs. 60%, *p* = 0.163; Table 1). However, discernible features such as bigger perivitelline space and granular cytoplasm were observed in long CAPA-IVM oocytes, indicating signs of aging.

To evaluate embryo development, 63 matured oocytes from the control and 34 matured oocytes from the long CAPA-IVM group were fertilized by piezo-drilled intracytoplasmic sperm injection (ICSI). Our data demonstrates a trend of compromised development in long CAPA-IVM with a lower cleavage (41% vs. 62% *p* = 0.050) and blastocyst rates (3% vs. 13% *p* = 0.114) compared to the control group. Out of 34 injected oocytes, only one embryo was able to form a blastocyst (Table 1).

### 2.2. Short CAPA-IVM Significantly Improved Maturation Rates and Embryo Development

In the next phase, we reduced the duration of pre-maturation culture to 6 h and assessed the effect on oocyte quality and developmental potential. For this purpose, a total of 546 oocytes (13 replicates) were cultured in both groups. Following IVM, short CAPA-IVM oocytes (*n* = 266) showed similar survival (63% vs. 56%, *p* = 0.131) yet significantly higher maturation rate (61% vs. 50%, *p* = 0.013) compared to the control (*n* = 280) (Table 2). Following IVM, mature oocytes were used to assess developmental competence and embryo genomics (seven replicates), proteomics (three replicates), and Ca oscillation patterns (three replicates).

After IVM, morphologically normal oocytes with an extruded polar body (69 short CAPA-IVM, 63 control; seven replicates) were fertilized by piezo-drilled ICSI to assess embryo developmental competency. A marked improvement in developmental potential was observed after short CAPA-IVM. In the short CAPA-IVM group, 56/69 (81%) injected oocytes cleaved on day 3, which was significantly higher compared to the control, i.e., 40/63 (63% *p* = 0.022). Similarly, a significantly higher number of embryos developed to blastocyst in short CAPA-IVM (20/69, 29%) when compared to the control group (9/63, 14% *p* = 0.041) (Table 2). Nonetheless, we noticed a comparable embryo growth rate in both groups, with an average day of blastocyst formation of 8.5 ± 0.246 in short CAPA-IVM and 8 ± 0.289 in control (*p* = 0.2633).

### 2.3. Equine Oocytes Demonstrate an Irregular Calcium-Releasing Pattern

**Figure 1:**
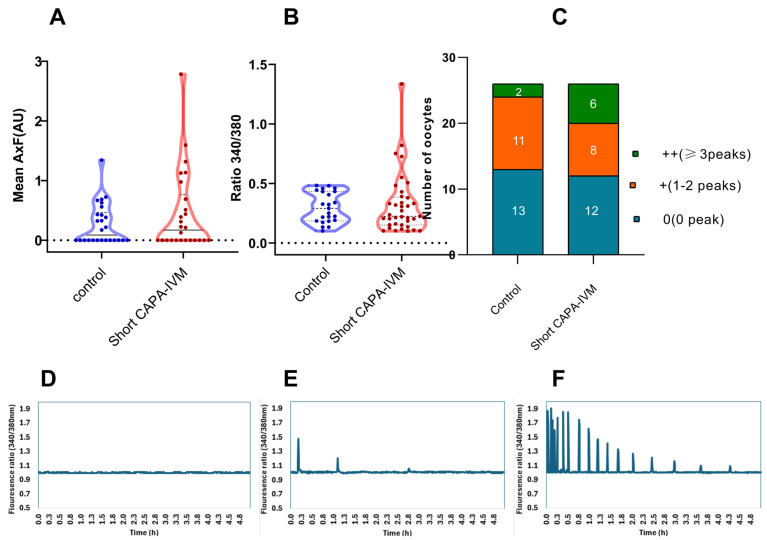
Live calcium imaging of the matured oocytes from control and short CAPA-IVM groups (data from three biological replicates): (**A**) calcium-releasing potential represented as the product of amplitude and frequency and expressed as arbitrary units (AU) (each dot represents one oocyte), (**B**) mean peak amplitude (each dot represents one calcium peak) (**C**) number of peaks produced by oocytes in each group. (**D**) 0 no peak, (**E**) 1–2 peaks, (**F**) ≥3 peaks.

Calcium imaging was performed after fertilization with a single motile sperm to observe the activation potential of in vitro-matured oocytes. Our data reveal that a higher number of oocytes in the short CAPA-IVM group were able to produce consistent calcium release patterns (higher frequency over time) (Figure 1C). However, no significant difference in calcium release was observed in the short CAPA-IVM compared to control (0.46 ± 0.68 vs. 0.26 ± 0.34, *p* = 0.5565) (Figure 1A). To investigate the discrepancy between the lower number of oocytes with calcium oscillations and the higher cleavage rate, we cultured the presumed zygotes following calcium imaging. Notably, by day 3, a higher proportion of zygotes reached the cleavage stage (79% and 50%) compared to the percentage of oocytes displaying calcium peaks (50% and 46%) in the short CAPA-IVM and control groups, respectively.

### 2.4. Genetic Analysis of Developed Embryos Shows Higher Euploidy Rates Across the Groups

**Figure 2:**
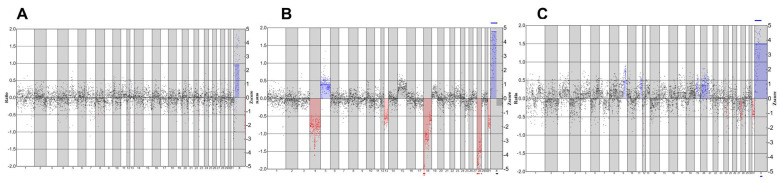
Representative SWGS profiles of equine blastocysts. Blue color indicates chromosomal addition, and red color indicates chromosomal deletions (**A**) Normal genetic profile. (**B**) An abnormal genetic profile with multiple additions and deletions. (**C**) Inconclusive genetic profile.

To further evaluate the effect of maturation culture on the chromosomal integrity of the embryos, we analyzed the developed blastocysts for copy number variations. Following analysis, we detected embryos with normal genetic profiles (Figure 2A), embryos with abnormal profiles (Figure 2B), and embryos with an inconclusive profile (Figure 2C). Shallow whole-genome sequencing data indicate a high euploidy rate in developed blastocysts, with 16/16 (100%) in short CAPA-IVM, 1/1 (100%) in long CAPA-IVM, and 21/23 (91%) in the control group. One blastocyst in short CAPA-IVM showed an inconclusive profile (Table 3).

### 2.5. The Proteome Is Maintained in In Vitro-Matured Oocytes Regardless of In Vitro Maturation Conditions

**Figure 3:**
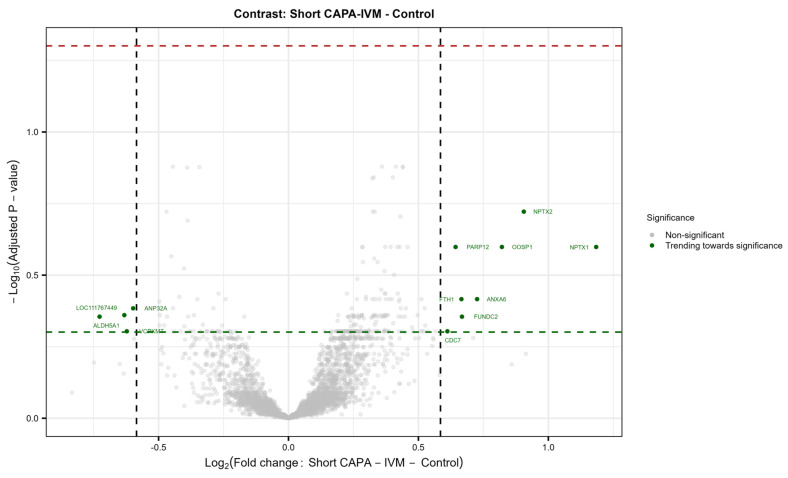
Volcano plot showing no differentially abundant proteins (DAPs) between matured oocytes from the control and short CAPA-IVM group (*p*. adj. ≤ 0.05, FC ≥ 1.5). Protein names indicated show IDs trending toward significance in the treatment group (Short CAPA-IVM). (*p* ≤ 0.05, FC ≥ 1.5). The green dashed line indicates the 0.05 *p*-value cutoff, whereas the red dashed line indicates the 0.05 adjusted *p*-value cutoff.

In total, 44 in vitro-matured oocytes (three replicates; control = 23; short CAPA-IVM = 21) were individually analyzed by mass spectrometry-based proteomics. The total number of reliably identified and quantified protein groups (hereafter referred to as proteins) in each sample is summarized in Figure S1B. A total of 4919 proteins were identified from single equine in vitro-matured oocytes across the entire dataset (Table S1). Our results show no significantly differentially abundant (DA) proteins in the short CAPA-IVM group compared to the control group. Furthermore, most of the variance in the dataset was correlated to the sample replicate rather than the treatment condition (Figure S1C,D). Nonetheless, we observed a trend of differential expression for certain proteins in the short CAPA-IVM group (Figure S2), mainly related to oocyte maturation fertilization (WEE2), oocyte activation (ANXA6), meiotic progression (CDC7), spindle–chromosomal stability (PARP12), and cellular stress management (FTH1) (Figure 3).

**Figure 4:**
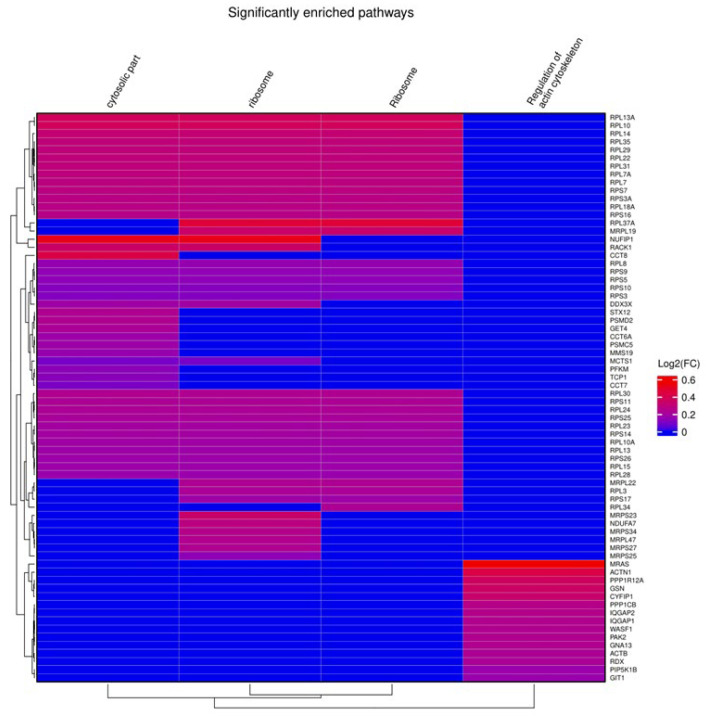
Gene set enrichment analysis (GSEA) of short CAPA-IVM oocytes compared to control oocytes. The figure shows GSEA results using a heat map, including the list of genes (right side of the heat map) involved in the different pathways (on top of the heat map).

From gene set enrichment analysis (GSEA) against Gene Ontology (GO) terms and pathways from the Kyoto Encyclopedia of Genes and Genomes (KEGG), we identified four enriched gene sets with positive enrichment scores in short CAPA-IVM, related to cytoskeleton regulation (Bta04810, FDR of 0.044), ribosomal function (GO:0005840, Bta03010, FDR of, respectively, 0.021 and 0.014), and cytosolic components (GO:0044445, FDR of 0.035) (Figure 4).

## 3. Discussion

Optimizing IVM protocols remains a key challenge in human and animal ART, particularly in enhancing oocyte developmental competence [4,15]. Our findings highlight the potential influence of pre-maturation culture duration on extended IVM culture and its impact on oocyte quality [43,44,45,46,47]. We observed that CNP-based CAPA-IVM holds the potential to improve equine oocyte quality and developmental potential. Moreover, proteome trends observed in this and recently published studies [48,49] offer new perspectives on optimizing the IVM protocol, both for horses and humans.

A 24 h pre-maturation in CAPA-IVM yielded a marked improvement in maturation without compromising oocyte survival. This increase in maturation rates has been demonstrated in bovine [50], murine [51], and human oocytes [26], where extended pre-maturation culture resulted in improved nuclear maturity. Nevertheless, despite improved maturation rates, we observed discernible aging-related morphological features such as granular cytoplasm and a large perivitelline space [52,53,54] in these oocytes, which ultimately resulted in poor embryo development. A trend towards compromised developmental potential was observed in the long CAPA-IVM group, as evidenced by lower cleavage rates (41% vs. 62%, *p* = 0.050) and blastocyst formation rates (3% vs. 13%, *p* = 0.114).

Our observation on the compromised oocyte morphology in long CAPA-IVM is further supported by a study on equine oocytes where longer pre-maturation duration resulted in compromised gap junctions in COCs [40]. Gap junction (GJ)-mediated communication with cumulus cells is crucial in supporting oocyte metabolism, compensating for its limited ability to metabolize glucose and uptake certain amino acids [55,56,57]. Furthermore, the passage of cyclic nucleotides via GJs safeguards prophase arrest in meiotically competent gametes, allowing for continued cytoplasmic growth and the acquisition of developmental competence [24,50,58,59,60]. Moreover, in porcine oocytes [61], it was demonstrated that prolonged IVM showed poor oocyte quality marked by high ROS levels, apoptosis, and compromised developmental competency compared to good-quality oocytes matured within standard maturation time.

It has been reported that a large perivitelline space in equine oocytes is associated with compromised developmental potential [62]. Previous investigations on human oocytes indicated that extended in vitro maturation culture was found to be associated with altered mitochondrial function, enhanced oxidative stress, and spindle abnormalities [63,64,65,66], which may lead to compromised development [66,67] and higher aneuploidy rates [68]. An equine study showed that prolonging IVM to 48 h significantly decreased cleavage and blastocyst rates [69]. These results demonstrate that while long CAPA-IVM may be beneficial in terms of improved maturation, the accompanying effect of oocyte aging affects the oocyte’s potential to support normal embryogenesis. Hence, it suggests the necessity of optimizing the timing of premature culture to improve cytoplasmic maturity without inducing ageing.

In the next setting, we reduced the duration of the pre-maturation culture to 6 h, as previously investigated on caprine [70], bovine [45], and equine oocytes [40]. As previously reported by Lodde et al. [40], a 6 h cilostamide-based pre-maturation helps maintaining open gap junctions and improves embryo quality in term of cells number per blastocyst. It has been shown in equine oocytes that GJ-based coupling is severely compromised if pre-maturation culture is extended beyond 10 h [40]. Nonetheless, no overall increase in blastocyst rate was reported with the use of cilostamide [40]. Our findings from the short CAPA-IVM condition indicate that a shorter pre-maturation culture duration offers an effective balance between promoting oocyte maturation and supporting subsequent embryo development. Specifically, we observed significantly improved maturation rates accompanied by enhanced embryonic development in the short CAPA-IVM group. These outcomes are consistent with previously published data in both caprine [70] and equine models [40], further supporting the utility of short pre-maturation protocols in optimizing IVM efficiency.

These results are consistent with the existing literature on human [43], murine [44], and bovine [45,46,47] IVM studies where shorter pre-maturation cultures improved embryo quality and developmental potential. This could be attributed to reduced accumulation of stress-related components and relatively better conserved mitochondrial function [64]. However, the exact duration of pre-maturation culture seems species-specific [15].

Calcium-imaging analysis reveals that oocytes exposed to short CAPA-IVM show a tendency for a higher calcium releasing potential (AU) than those matured under control conditions. Previously, only few studies have performed live calcium imaging to determine the calcium-releasing potential of equine IVM oocytes. Overall, the calcium oscillation patterns were inconsistent and were only observed in a small fraction of injected oocytes [71,72]. This may partially explain the suboptimal embryo outcomes frequently observed in equine ARTs, as aberrant calcium-releasing mechanisms can impair fertilization efficiency and lead to early embryonic arrest [73]. Calcium signaling is essential for key developmental processes, including meiotic progression, pronuclear formation, maternal mRNA recruitment, and early embryogenesis [74]. However, a higher number of oocytes were activated even without detectable calcium oscillations [71], comparable with our results showing a higher cleavage rate (79% and 50%) on culturing presumed embryos after calcium imaging, despite a lower number of oocytes showing detectable calcium release (50% and 46%) in the short CAPA-IVM and control groups, respectively. This may represent a species-specific characteristic of equine oocytes, where not all oocytes activate immediately. Notably, injection of the same equine sperm into mouse oocytes resulted in more consistent and robust calcium release, suggesting the delay may be inherent to the oocyte rather than the sperm [72]. To further test the possible insufficient delivery of oocyte activation factor, Bedford et al. [72] permeabilized the sperm membrane by sonication or treatment with Triton-x before ICSI. Contrary to improved calcium oscillations in other species [75,76,77], it does not improve the Ca^2+^ pattern, suggesting the oocyte’s inability to process the oocyte activation factor delivered by spermatozoa.

Shallow whole-genome sequencing of the developed equine blastocysts showed high euploidy rates across different maturation conditions, with 100% in short CAPA-IVM and long CAPA-IVM and 91% in control groups. These results indicate that the prolonged maturation culture in CAPA-IVM does not affect chromosomal stability, which is similar to previously reported data on human PCOS patients, where CAPA-IVM did not affect euploidy rates [27]. The occurrence of high euploidy rates in equine blastocysts is comparable with a recently reported study by De Coster et al. 2024 [78] where 12 out of 14 analyzed blastocysts showed normal euploid profiles. However, in the same study, it was shown that five of six arrested in vitro-produced (IVP) embryos exhibited chromosomal abnormalities, which could be the reason for their embryonic arrest. Chromosomal integrity is crucial for embryo viability and positive pregnancy outcomes, as aneuploidy is one of the major reasons for implantation failure and pregnancy loss across various species [79,80]. Altogether, it can be assumed that high euploidy rates in developed blastocysts could be a contributing factor for satisfactory pregnancy, i.e., ~70% [38], and live birth rates, i.e., ~60% [81], in equine breeding programs. Therefore, CAPA-IVM’s ability to retain an overall high euploidy rate further encourages its utility in clinical OPU-ICSI programs to improve ART outcomes.

To further develop a deeper understanding of the molecular mechanisms of extended IVM, for the first time, we performed single-oocyte proteomics on in vitro-matured equine oocytes. The data retrieved from this analysis comprehensively describe the proteomic landscape of these oocytes. After filtering, a total of 4919 proteins were reliably identified and quantified in a single equine in vitro-matured oocyte. We noticed that the oocyte proteome does not undergo significant changes at the individual protein level, though four significantly enriched gene sets were observed in short CAPA-IVM oocytes. Moreover, our proteome study revealed a trend of differentially expressed proteins in equine oocytes following short CAPA-IVM, mainly associated with oocyte maturation, activation, and developmental competence. Since we only compared pre-selected matured oocytes from different in vitro maturation groups, proteomic differences leading to failed maturation will not be apparent. Additionally, given that the oocyte is transcriptionally silent during the maturation process, transcripts produced earlier might not be fully translated at the M-II stage. Hence, proteomic differences could become more pronounced in later phases of embryo development, explaining the observed discrepancy in development outcomes. Therefore, proteome analysis of subsequent embryos may offer a more comprehensive understanding of CAPA-IVM’s impact on oocyte potential. Furthermore, it would be interesting to compare the proteomes of in vitro-matured oocytes to those of in vivo-matured ones, as a “golden standard” or positive control, together with proteomic analysis of subsequently developed embryos.

Our GSEA revealed four significant enrichments of pathways associated with ribosomal function, cytosolic components, and cytoskeletal organization in the short CAPA-IVM oocytes, suggesting the presence of a molecular environment conducive to cytoplasmic maturation. The enrichment of cytoskeletal pathways (Bta04810, FDR = 0.044) indicates enhanced spindle organization and actin filament remodeling, which are involved in oocyte polarity, chromosome alignment, and asymmetric division [82]. Notably, PPP1R12A, a regulator of myosin phosphatase and a member of this gene set, is implicated in CNP-cGMP-PKG signaling, a key pathway in maintaining oocyte arrest [83]. The presence of PPP1R12A in this enriched gene set suggests that CAPA-IVM may influence cytoskeletal arrangement via cyclic nucleotide-mediated pathways, essential for better structural integrity and spindle–chromosome complex [83].

Likewise, the upregulation of ribosomal function-related pathways (GO:0005840, Bta03010, FDR = 0.021 and 0.014, respectively) suggests that short CAPA-IVM supports translational activity required for maternal mRNA processing and protein synthesis during maturation [84]. Since oocytes rely on stored maternal transcripts to drive early embryonic development, an effective ribosomal activity might be instrumental for the improved developmental potential observed in our study [84]. The enrichment of cytosolic component-related pathways (GO:0044445, FDR = 0.035) further supports the idea that intracellular organization, protein stability, and metabolic regulation are improved in short CAPA-IVM oocytes, creating a more supportive environment for maturation and fertilization.

Overall, short CAPA-IVM demonstrates a promising approach to synchronize nuclear and cytoplasmic maturation while limiting stress-induced damage. Future research should focus on validating these findings through functional assays to understand their effect on oocyte biology in a wider context. Furthermore, comparative single-oocyte omics analyses across species could provide valuable insights into conserved and species-specific mechanisms, facilitating the refinement and enhancement of in vitro maturation protocols.

## 4. Materials and Methods

**Figure 5:**
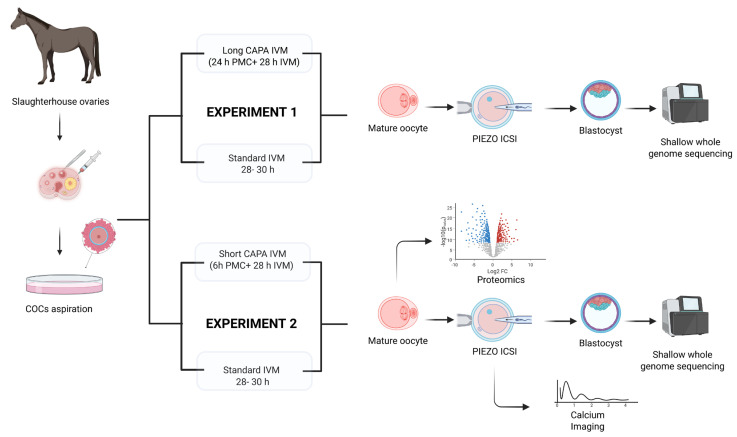
Experimental design for evaluating CAPA-IVM in equine oocytes in two different settings: Experiment 1 compared long CAPA-IVM with standard IVM but was discontinued due to its negative impact on oocyte morphology and embryo development. Experiment 2 compared short CAPA-IVM with standard IVM. Mature oocytes were further analyzed for embryo development after piezo ICSI (intracytoplasmic sperm injection), calcium releasing potential, and by single-oocyte proteomics. Shallow whole-genome sequencing was performed on developed blastocysts to detect chromosomal aberrations.

The experimental design followed for this study is shown in Figure 5.

### 4.1. Collection of Equine COCs

Equine ovaries were collected from a local abattoir in Belgium and transported to the laboratory in an insulated box at ambient temperature within 1 h of collection. Ovaries were cleaned by removing surrounding tissue and washed twice with prewarmed normal saline (38.2 °C; NaCl 0.9%). All follicles ranging from 5 to 30 mm were aspirated using a 16-gauge needle attached to a vacuum pump (Craft Suction Pump, Rocket Medical, Washington, UK) (100 mm Hg), scraped with the aspirating needle, and flushed with prewarmed flushing medium (Equiplus, Minitube, Tiefenbach, Germany). The aspirated fluid was collected in sterilized glass bottles and searched under a stereomicroscope (Olympus SZX7^®^, Olympus Corp. Shinjuku, Tokyo, Japan) for the presence of COCs at room temperature (22 °C). During recovery, COCs were washed and kept in prewarmed TCM-199 with Hank’s salts (38.2; Gibco, Life Technologies, Merelbeke, Belgium) supplemented with 10% *v*/*v* FBS (Gibco, Life Technologies, Merelbeke, Belgium) and 0.1% gentamycin *v*/*v* (Sigma-Aldrich, Bornem, Belgium) before transferring to the maturation culture. Only COCs with compact and fully surrounding cumulus cells were considered for further IVM.

### 4.2. In Vitro Maturation and Fertilization

The recovered COCs were either placed in a pre-maturation culture with IVM medium containing 200 nM CNP (Bio-Techne, Dublin, Ireland) for 6 h (short CAPA-IVM) or 24 h (long CAPA-IVM) before IVM, or directly transferred to IVM culture (Medium 199 with Earl’s salts (Gibco, Life Technologies, Merelbeke, Belgium), supplemented with 10% FBS (*v*/*v*) (Gibco, Life Technologies, Merelbeke, Belgium), 9.8 μg/mL follicle-stimulating hormone, and 1.96 μg/mL luteinizing hormone (Stimufol, Reprobiol, Ouffet, Belgium)) as a control. In vitro maturation was performed in groups of 10–20 COCs in 500 μL maturation medium under oil (CooperSurgical, Venlo, The Netherlands) at 38.2 °C in 5% CO_2_-containing air for 28–30 h. Denudation was performed by enzymatic treatment [0.1% bovine hyaluronidase (Sigma-Aldrich, Bornem, Belgium) in TCM-199 with Hank’s salts (Gibco, Life Technologies, Merelbeke, Belgium) supplemented with 10% FBS (Gibco, Life Technologies, Merelbeke, Belgium) and 0.1% gentamycin (Sigma-Aldrich, Bornem, Belgium))] and mechanical stripping using a STRIPPER pipettor and 170 μm and 135 µm capillaries (MXL3-STR-CGR, MXL3-175 and MXL3-135; Cooper Surgical, Trumbull, CT, USA). The nuclear maturity of the oocyte was assessed by the presence of the first polar body.

For oocyte fertilization, ICSI was performed as described previously [78,85]. Briefly, a small piece of straw with frozen semen from a proven fertile stallion was thawed in 1 mL G-MOPS (38.2 °C; Vitrolife, Londerzeel, Belgium) and centrifuged (400× *g*/3 min; 23 °C). After the first centrifugation step, the supernatant was discarded, and the pellet was resuspended in 1 mL G-MOPS. After the second centrifugation step, the supernatant was discarded, and the pellet was resuspended in 200 μL G-MOPS. At the time of ICSI, a small volume (1 μL) of the resuspended sperm was added to a 5 μL droplet of 7% polyvinylpyrrolidone (CooperSurgical, Venlo, The Netherlands). Metaphase (M-II) oocytes were injected with piezo-drilled ICSI (PrimeTech, Nakamukaihara Tsuchiura Ibaraki, Japan; speed 3–4, intensity 6–8), and presumptive zygotes were cultured for 7–10 days in 20 μL droplets of DMEM/F-12 (Gibco, Life Technologies, Merelbeke, Belgium) with 10% FBS and 0.1% gentamycin under oil at 38.2 °C in a humidified atmosphere of 5% O_2_, 5% CO_2_, and 90% N_2_. The cleavage rate was evaluated on day 3 after ICSI, and blastocyst formation was monitored from day 7 to day 10 post-ICSI.

### 4.3. Calcium Imaging of In Vitro-Matured Oocytes

Calcium imaging was performed on equine in vitro-matured oocytes, as described previously [86]. Briefly, oocytes were exposed to 7.5 μΜ fura-2-AM (Teflabs, Austin, TX, USA) in an embryo culture medium for 30 min under standard culture conditions. Then, oocytes were injected with a single sperm via piezo-drilled ICSI using an inverted microscope(Olympus IX73; Olympus Shinjuku, Tokyo, Japan). The selected spermatozoa were demembranated with 0.2% lysolecithin (Instruchemie, Delfzijl, The Netherlands) for 1 min to ensure efficient delivery of the oocyte activation factor. ICSI was performed within 30 min, and oocytes were transferred to an inverted epifluorescence microscope (Olympus IX71, Olympus Soft Imaging Solutions GmbH, Antwerpen, Belgium) in a glass-bottom dish (MatTek Corporation, Ashland, MA, USA) under standard culture conditions.

Calcium release was recorded for five consecutive hours, with a 10× objective and a filter switch (Lambda DG-4 filter switch, Sutter Instrument Company, Novato, CA, USA) to provide excitation alternating between 340 and 380 nm. Calcium data were analyzed using Clampfit 10.2 software (Axon Laboratories, Molecular Devices UK Ltd., Winnersh, UK). The total amount of calcium released (in arbitrary units (AU)) was calculated as the product of the mean amplitude (maximum fluorescence intensity per peak) per mean frequency (number of calcium spikes) of all oocytes injected per condition (including the oocytes showing no calcium peaks).

### 4.4. Genetic Analysis of Developed Embryos

Embryos reaching the blastocyst stage were immediately collected for genetic analysis and later analyzed by shallow whole-genome sequencing as described [87]. Briefly, the zona pellucida of each blastocyst was removed by treating with EmbryoMax^®^ Acidic Tyrode′s Solution (Merck Life Science, Overijse, Belgium) for 1 min. After zona pellucida removal, blastocysts were thoroughly washed and collected in Dulbecco’s phosphate-buffered saline (DPBS) and stored at −20 °C until processing for genetic analysis. Whole-genome amplification (WGA) was performed with the SurePlex DNA amplification kit (Rubicon Genomics Inc., Ann Arbor, MI, USA). DNA was fragmented to ~200 bp using a M220 Focused ultrasonicator Instrument (Covaris, Woburn, MA, USA), and subsequent library preparation was performed with the NEXTflex™ Rapid DNA-Seq Library Prep Kit for Illumina Sequencing (Bioo Scientific, Uden, The Netherlands). Agencourt AMPure XP beads (Beckman Coulter, Suarlée, Belgium) were used for purification. Template preparation was performed on the cBot™ System (Illumina, San Diego, CA, USA), using 2.5 nM of equimolar pooled libraries, and sequencing was performed on a Hiseq3000 sequencer (Illumina, San Diego, CA, USA). CNV detection data was analyzed using the WisecondorX and Vivar software [88,89].

### 4.5. Single-Oocyte Proteome Sample Preparation

Single-oocyte proteomics was performed as described [90]. In three replicates, individual in vitro-matured oocytes were sampled in 2 µL of DPBS in a twin.tec^®^ PCR Plate 384 LoBind^®^ (Eppendorf 0030129547, Hamburg, Germany) and stored frozen at −80 °C until further processing. Samples were lysed by three thaw–freeze cycles at 80 °C followed by −70 °C for 15 min each, with centrifugation at 1500 *g* for 10 s after each thawing step. During each incubation step, plates were tightly sealed using plastic plate seals (Diversified Biotech, Dedham, MA, USA). After the first thawing step, 1 µL of 15% LC-MS-grade DMSO (Thermo Scientific, Rockford, IL, USA) was added to each sample. Proteins were then digested overnight at 37 °C in 3 µL of digestion buffer (100 mM triethylammonium bicarbonate (TEAB) (Merck, Darmstadt, Germany), 0.1% n-Dodecyl-B-D-maltoside (Sigma-Aldrich, Steinheim, Germany), 0.02% ProteaseMAX (Promega, Madison, WI, USA), 13.33 ng/µL Trypsin/Lys-C (Promega, Madison, WI, USA)). Subsequently, samples were acidified with 1 µL of 5% trifluoracetic acid (TFA) (Biosolve, Dieuze, France). Samples were transferred to a twin.tec^®^ PCR Plate 384 LoBind^®^ using protein low-binding p10 tips (Socorex, Langenhagen, Germany); in doing so, sample volumes were estimated. Sample volumes were brought to 15 µL using 0.1% TFA. The plate was tightly sealed using an aluminum plate seal (Excel Scientific, Victorville, CA, USA) and stored at −70 °C until data acquisition at the VIB proteomics core facility.

### 4.6. Proteomics Data Analysis

Raw data files were searched using DIA-NN v1.9.2. The search engine was supplied with the equine reference proteome (*Equus caballus*: UP000002281 with 69,434 entries) for library generation, allowing for one missed cleavage using Trypsin/P as the protease and up to two variable modifications, with N-terminal methionine excision, methionine oxidation, and N-terminal acetylation set as variable modifications. Peptide length and charge ranges were set to 7–30 and 1–4, respectively. Precursor and fragment ion ranges were set to 400–1000 and 100–1700, respectively. Precursors were filtered at 1% FDR, with quantUMS (high precision), cross-run retention time-based normalization, and match between runs enabled.

Downstream data analysis was performed with R version 4.4.0 within the RStudio environment version 2024.09.0+375. Statistical analysis was performed using the MSqRob2 and QFeatures packages [42,43]. Plotting was performed using the ggplot2 package [44]. Gene set enrichment analysis (GSEA) was performed using the WebGestaltR package [45]. Briefly, only precursors identified in at least five control and five treated samples, proteins for which at least two peptides were identified, and which were identified in each technical batch, were considered for statistical modelling. Precursor quantities were log_2_-transformed, median-normalized, and then aggregated into protein abundances using the median polish procedure implemented in MSqRob2. Differential expression analysis was performed using MSqRob2. *p*-values were adjusted using the Benjamini–Hochberg method with an FDR of 0.05. GSEA was performed using the -log_2_(fold change) × sign(fold change) as a ranking metric, with an FDR of 0.05. The queried database consists of KEGG pathways [91] and the biological process, cellular component and molecular function gene ontologies of a size between 50 and 200 genes. Mass spectrometry raw data is available via the PRIDE data repository (Accession number PXD060584; reviewer username: reviewer_pxd060584@ebi.ac.uk, reviewer password: IPzt5yEKzlBb).

### 4.7. Statistical Analysis

The chi-square (χ^2^) test was applied to compare the categorical variables expressed in percentages. The average day of blastocyst formation and calcium-releasing potential (AU) were compared among groups using the Mann–Whitney U test. *p*-values < 0.05 were considered as statistically significant. Statistical analysis was performed using GraphPad Prism version 10.0.2 (GraphPad Software, Boston, MA, USA).

## 5. Conclusions

Our research encompasses crucial implications for optimizing IVM systems in both human and equine ARTs. The reciprocal knowledge from both human and equine models in ART research has substantial potential to accelerate progress in both fields. CAPA-IVM, particularly with optimal pre-maturation culture duration, offers a promising approach for enhancing oocyte maturation and embryo development in equine ART. The variable effect of short and long CAPA-IVM on oocyte quality and developmental potential highlights the importance of pre-maturation culture duration to obtain optimal outcomes. The proteomic profiles of IVM MII oocytes from both groups shed light on subtle differences which may impact further development. The high euploidy rates across different culture conditions further highlight the ability of CAPA-IVM as an alternative to traditional IVM, improving oocyte developmental potential with retained genetic integrity.

## Figures and Tables

**Table 1:** Survival, maturation, cleavage, and blastocyst rates in long CAPA-IVM and control group. Data is the observation of three replicates. Asterisk (*) indicates a statistically significant difference compared to the control group (*p* < 0.05).

	Control	Long CAPA-IVM	*p*-Value
COCs	138	61	
Survival rate (%)	83/138 (60%)	43/61 (70%)	0.163
Maturation rate (%)	70/138 (51%)	42/61 (69%) *	0.017
Oocytes injected	63	34	
Cleavage rate (%)	39/63 (62%)	14/34 (41%)	0.050
Blastocyst rate (%)	8/63 (13%)	1 (3%)	0.114

**Table 2:** Oocyte and embryo outcomes in short CAPA-IVM and control group (maturation and survival rates =13 replicates; cleavage and blastocyst rates = 7 replicates). Asterisk (*) indicates a statistically significant difference compared to the control group (*p* < 0.05).

	Control	Short CAPA-IVM	*p*-Value
COCs	280	266	
Survival rate (%)	158/280 (56%)	167/266 (63%)	0.131
Maturation rate (%)	141/280 (50%)	162/266 (61%) *	0.013
Oocytes injected	63	69	
Cleavage rate (%)	40/63 (63%)	56/69 (81%) *	0.022
Blastocyst rate (%)	9/63 (14%)	20/69 (29%) *	0.041

**Table 3:** Euploidy rate in developed blastocysts across different maturation groups.

	Control	Short CAPA-IVM	Long CAPA-IVM
Euploidy rate (%)	21/23 (91%)	16/16 (100%)	1/1 (100%)
Inconclusive profiles	0	1	0

## Data Availability

All data generated or analyzed during this study were included in the manuscript and its Supplementary Information Files. Raw data are available from the corresponding author upon reasonable request. The mass spectrometry proteomics data and analysis scripts have been deposited to the ProteomeXchange Consortium via the PRIDE partner repository with the dataset identifier PXD060584 (https://www.ebi.ac.uk/pride/archive accessed on 8 June 2025).

## Supplementary Materials

The following supporting information can be downloaded at: https://www.mdpi.com/article/10.3390/ijms26125495/s1.
