# Supplementary material for: Biphasic CAPA-IVM Improves Equine Oocyte Quality and Subsequent Embryo Development Without Inducing Genetic Aberrations
Source: Int J Mol Sci. 2025 Jun 8;26(12):5495. doi: 10.3390/ijms26125495 (PMC12192595; doi:10.3390/ijms26125495)
Supplement: Supplementary file 1 [file ijms-26-05495-s001.zip › supplementary.pdf]

Supplementary materials and methods:

Liquid chromatography-tandem mass spectrometry analysis

Proteomic samples were run at the VIB proteomics core facility in data-independent parallel accumulation serial fragmentation (diaPASEF) mode on an UltiMate 3000 LC-system (Thermo Scientific, Bremen, Germany) in-line connected to a timsTOF Ultra mass spectrometer (Bruker, Bremen, Germany). From each sample, 14.5 µL was injected and analyzed with a 30-minute gradient using a trap-and-elute workflow. Trapping was performed at 20 µL/min for 2 min in loading solvent A (0.1% TFA in water/acetonitrile (ACN; Fisher Chemical™, Loughborough, UK)) on a PepMap™ Neo Trap column (Thermo scientific, 300 µm internal diameter (I.D.), 5 µm beads), followed by separation on an Aurora Gen3 Elite column (15cm x 75 µm I.D., 1.7 µm C18 beads, Ionopticks, Fitzroy, Australia), heated to 50°C. Peptides were eluted by a linear gradient starting at 0.5% MS solvent B (0.1% FA in water/ACN 20:80 (v/v)), increasing to 37.5% MS solvent B at 15 min, 55% after 19 min, 70% after 20 min, followed by a wash for 2.5 min and re-equilibration with 99.5% MS solvent A (0.1% FA in water). The flow rate was decreased from 250 nL/min to 100 nL/min at 10 min and increased again to 250 nL/min at 20 min.

Eluting peptides were measured in positive polarity with a full-scan range of m/z 100 to 1700. The trapped ion mobility spectrometry (TIMS) module was operated at a fixed duty cycle close to 100%, a ramp and accumulation time of 100 ms, ranging from $1/K_0$ =0.64 Vs/cm^2 to 1/K0 =1.50 Vs/cm^2. Collision energy was linearly ramped as a function of the inverse mobility from 20 eV at $1/K_0$ = 0.60 Vs/cm^2 to 59 eV at $1/K_0$ = 1.60 Vs/cm^2. A diaPASEF mass range of 400 to 1000 m/z was used in a mobility range of $1/K_0$ = 0.64 Vs/cm^2 to $1/K_0$ = 1.37 Vs/cm^2 using a window size of 25 m/z according to Supplementary Table S1, resulting in a total cycle time of 0.96 s.

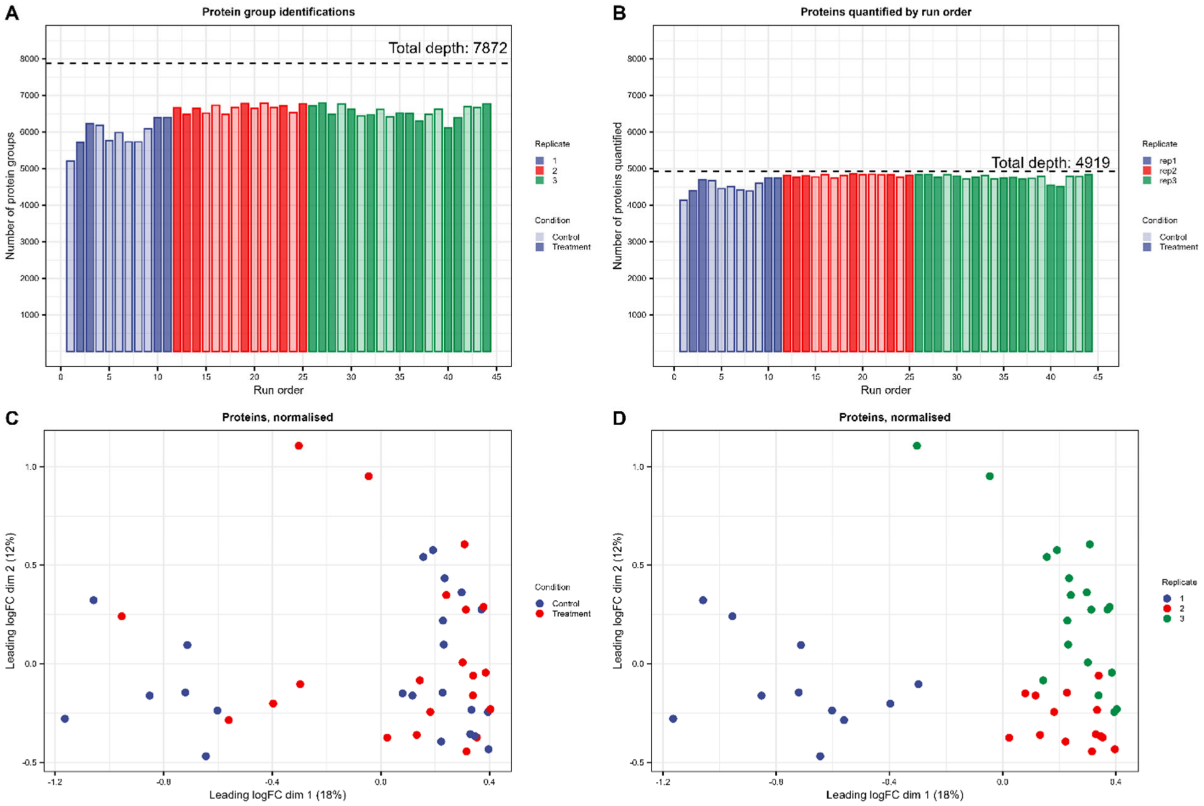

Figure S1: Protein groups identified and quantified across the entire dataset (A) before data filtering, (B)after filtering. MDS plots (C, D) show samples clustered by replicate rather than the treatment.

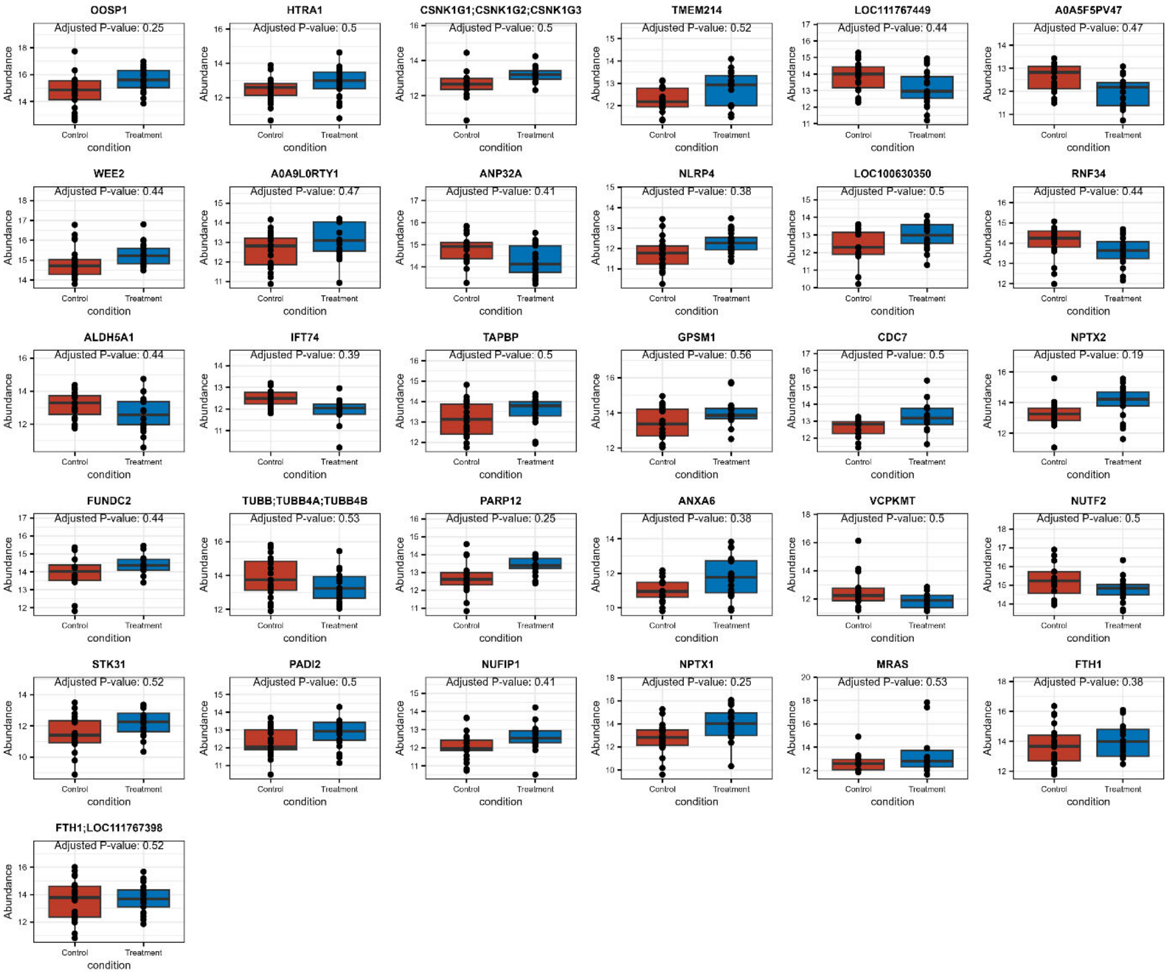

Figure S2. Abundance values of proteins trending toward significance between control and CAPA-IVM oocyte groups. Each panel represents an individual protein and black dots represent individual data points per biological replicate.
